# miR-425-5p Acts as a Molecular Marker and Promoted Proliferation, Migration by Targeting RNF11 in Hepatocellular Carcinoma

**DOI:** 10.1155/2020/6530973

**Published:** 2020-10-16

**Authors:** Dan Rao, Songmei Guan, Junwei Huang, Qing Chang, Shigang Duan

**Affiliations:** ^1^Department of Hepatobiliary Surgery, Chongqing Ninth People's Hospital, Chongqing 400700, China; ^2^Clinical Pharmacy, Chongqing Ninth People's Hospital, Chongqing 400700, China

## Abstract

Hepatocellular carcinoma (HCC) is one of the most common and dangerous malignant tumors in China, which causes a large number of deaths every year. MicroRNAs (miRNAs) dysfunction contributes to the malignant progression of tumors. The aim of our study was to investigate the relationship between the biological role of miR-425-5p and malignant progression of HCC. Our results showed that miR-425-5p expression was significantly upregulated in HCC tissues and closely related to the poor prognosis of HCC patients. The knockdown of miR-425-5p inhibited cell proliferation and migration. Further, we identified RNF11 as the downstream target gene of miR-425-5p. In addition, the rescue experiments showed that the upregulation of RNF11 could rescue the inhibitory effect of miR-425-5p on HCC. In general, miR-425-5p as an oncogene promotes the malignant development of HCC via RNF11 and serves as a molecular target for predicting the prognosis of HCC patients.

## 1. Introduction

Hepatocellular carcinoma (HCC) is the third most common malignant tumor with mortality after gastric cancer and esophageal cancer [1, 2]. Like other cancers, the onset of HCC is a complex process with multiple factors and multiple steps, which is affected by both environmental and genetic factors. Epidemiological and experimental research data have shown that HBV and HCV infection, aflatoxin B1, alcohol, liver cirrhosis, and sex hormones are related to the onset of HCC, and HBV and HCV infection are the most important factors [3, 4]. The existing research results indicate that the X protein of HBV plays an important role in the occurrence and development of liver cancer. X protein is a multifunctional protein encoded by the HBV genome. Its functions include tyrosine phosphokinase activity, p53 protein binding activity, transcription activation, Src kinase activation, and DNA excision repair system inhibition [5]. In addition, the activation or amplification of Ras and Myc genes plays an important role in the proliferation of HCC cells [6]. Ras-GTPase and its downstream mitogen-activated protein kinase effector pathways positively regulate the cell cycle in a variety of cells [6, 7]. Wnt signaling pathway and TGF-*β*/Smads signaling pathway are signal transduction chains with extensive functions. Mutations in this pathway exist in HCC cells, which promote the malignant growth of liver cancer [8]. However, the biological characteristics of HCC are that the disease is concealed and the prognosis is poor, which brings a heavy burden to the individual and society [9, 10]. Therefore, it is very important to clarify the molecular mechanism of the occurrence and development of HCC and find effective target genes for molecular therapy.

The role of microRNAs (miRNAs) in tumorigenesis and development has been recognized by scientists. A large number of studies have found the expression and dysfunction of miRNAs in multiple malignant tumors including gastric cancer, colon cancer, and bladder cancer [11–13]. In oral carcinoma, miR-31 causes the stability of mitochondria and membrane potential through SIRT3 and promotes the occurrence of oxidative stress, resulting in an increase in glycolysis in tumor cells, ultimately promoting the malignant development of tumors [14]. A study also found that miR-29c-3p promotes the malignant development of HCC by regulating the methylation of LATS1 caused by DNMT3B and inhibiting the anticancer function of the Hippo signaling pathway [15]. In addition, the miRNA-140-5p regulation of TGFBRI and IGF1R expressions results in the abnormal expression of downstream target gene SMAD2/3 and p-AKT and causes the malignant development of Wilms' tumor [16]. Recently, a study has found that the high expression of miR-425-5p can promote the malignant development of gastric cancer by regulating the expression of CYLD [17]. However, the biological role of miR-425-5p in HCC is still unclear, and its role in HCC deserves our in-depth study.

RNF11 gene is a key member of the E3 ubiquitin ligase family [18]. Protein is precisely balanced by de novo synthesis, posttranslational modification, and degradation. 80% of the proteins in cells are destroyed by ubiquitin of the ubiquitin enzyme system [19]. RNF11 has a dominant position in the ubiquitination signal pathway. The function of RNF11 is very powerful, covers a wide range, and is very important to the body [20]. In the promoter region of mouse RNF11, three conserved ETS1 transcription factor binding sites were found. Among them, ETS1 is produced by many cells and is necessary for embryonic development, blood vessel formation, hormone production, lymphocyte production, and activation of immune cells, and ETS1 is activated by the action site of RNF11 bound to the ETS1 site and regulate transcription, so that RNF11 will work together with ETS1 at the transcription level of bone cells to play a regulatory role [21, 22]. RNF11 can increase the TGF-*β* signal starting from the receptor level. RNF11 can directly connect with SMAD4, and SMAD4 is a common Smad that interacts with TGF-*β*. RNF11 can enhance the transcriptional activity of SMAD4 and SMAD2-SMAD4 transcriptional complexes, and then play an important role in regulating the pathway [23]. However, there are few studies on RNF11 in HCC, and its specific role is still unknown.

In this study, we detected the expression of miR-425-5p in HCC samples and analyzed the relationship between miR-425-5p and poor prognosis of patients in HCC. In addition, the biological effects of miR-425-5p on HCC cells were confirmed by in vivo and in vitro. Important, we further analyzed the downstream target genes of miR-425-5p and its role in the malignant progression of HCC. Therefore, we aim at demonstrating whether miR-425-5p contributes to the diagnosis and treatment of HCC.

## 2. Materials and Methods

### 2.1. HCC Samples and Clinical Data

A total of 105 samples of HCC patients (HCC tumor tissue and paired adjacent tissue) were collected. These samples were retrospectively obtained from April 2008 to July 2013 at the Department of Hepatobiliary Surgery, Chongqing Ninth People's Hospital, Chongqing, China. All patients with HCC in this study signed an informed consent form. This study was approved by the Ethics Committee of the Chongqing Ninth People's Hospital and was conducted in accordance with the Declaration of Helsinki. All HCC samples were snap-frozen with liquid nitrogen for mRNA and protein assessment.

### 2.2. Cell Culture and Transfection

In the study, 2 HCC cell lines (HepG2 and Huh-7) were obtained from Shanghai Cell Bank of the Chinese Academy of Sciences (Shanghai, China). All cell lines were cultured in high-glucose DMEM (Gibco, USA) with 10% fetal bovine serum and 100 units/ml penicillin and streptomycin (Gibco, USA) and incubated at 37°C in a 5% CO2.

Cells were transfected with pcDNA3.1-RNF11, pre-miR-425-5p (miR-425-5p), and miR-425-5p inhibitor from GenePharma ((Shanghai, China). The cells were transfected with Lipofectamine 3000 reagent (Invitrogen, USA) according to the manufacturer's instructions.

### 2.3. Quantitative Real-Time Polymerase Chain Reaction (RT-qPCR)

Total RNA was extracted from HCC tissues and cell lines by TRIzol reagent (TaKaRa, Japan). Reverse-transcribed complementary DNA was used by PrimeScript RT Reagent (TaKaRa, Japan). Then, RT-qPCR was performed with PrimeScript® RT Reagent Kit (TaKaRa) using a LightCycler system (Roche). U6 was used as an internal control for microRNA, and GAPDH was used for mRNA expression. Relative RNA expression levels were calculated by the 2^-*ΔΔ*CT^ method. The RT-qPCR primes are as follows: miR-425-5p F: 5′- TGC GGA ATG ACA CGA TCA CTC CCG-3′, R: 5′- CCA GTG CAG GGT CCG AGG T-3; RNF11 F: 5′-CCT ATC CTC TGC GCT ATT CG-3′, R: 5′-GCC ATA TCA GGA GCA AGT CC-3; GAPDH F: 5′- AGG TCG GAG TGA ACG GAT TTG-3′, R: 5′- ACC ATG TAG TGG AGG TCA ATG AAG-3; U6 F: 5′-CTC GCA TTG GCA GGA CTT ATA CT-3′, R: 5′-AAT CGT CAC GAA TCT GTG AGT C-3′.

### 2.4. Western Blot

Total protein was isolated from HCC tissue and cell lines by protein extraction buffer (RIPA lysis buffer) and use BCA protein assay kit (Beyotime, Haimen, China) to determine the extracted protein concentration. The total protein was resolved by 10% sodium dodecyl sulfate-polyacrylamide gel electrophoresis (SDS-PAGE). Then, block the membranes for 1 hour at 37°C with 5% nonfat powdered milk. The membranes were probed at 4°C overnight with anti-RNF11 (1 : 1000, ab154831, Abcam) and anti-GAPDH (1 : 5000, ab181602, Abcam). Next, the membranes were incubated with the appropriate secondary antibodies. Finally, the chemiluminescence detection system was used to analyze protein expression.

### 2.5. Cell-Counting Kit-8 Assay

The CCK8 assay was used to determine the cell viability in the HCC cells. Transfected cells are seeded on a 96-well plate; cells were processed for the CCK8 assay according to the manufacturer's instructions.

### 2.6. Wound Healing Assay

Cells were inoculated on a 6-well plate. The cell monolayer was scratched with a pipette tip (1 ml) to generate 3 scratch wounds after the cells were full, and suspended cells were washed off with PBS. The distance between the wound sides was measured with a microscope.

### 2.7. 5-Ethynyl-2′-Deoxyuridine (EdU) Assay

The transfected cells were inoculated into a 96-well plate. Cells were immobilized with 4% polyformaldehyde and nucleus perforation with 0.5% Triton X-100 solution. According to the manufacturer's instructions, cells were incubated with EdU (50 *μ*M), 1 × ApolloR reaction cocktail (100 *μ*l), and 1 × Hoechst 33342 (100 *μ*l) for 30 min. Cell proliferation was analyzed using the mean number cells.

### 2.8. miR-425-5p Target Prediction

There are three prediction databases, including TargetScan (http://www.targetscan.org), Oncomir (http://www.oncomir.org/), and miRWalk (http://mirwalk.umm.uni-heidelberg.de/), were used to predict miRNA targets and conserved sites bound by miR-425-5p.

### 2.9. Luciferase Reporter Assay

The wild-type RNF11-3′UTR (WT) interacted with miR-425-5p and mutant RNF11-3′UTR (MUT) predicted target sites were synthesized and inserted into the pMIR-REPORT vector (Obio Technology, China). These constructs were transfected with inhibitor NC or miR-425-5p inhibitor into HCC cells. The luciferase assays were performed using the Dual-Luciferase Reporter Assay kit (Promega) after 48 h.

### 2.10. Statistical Analysis

All data were expressed as mean ± SD and analyzed using SPSS 22.0 software (SPSS Inc., Chicago, IL, USA) or GraphPad Prism version 7.0 (CA, USA). The differences were analyzed by one-way analysis of variance, followed by the Newman-Keuls test, were used to evaluate the differences between groups, and repeated measures analysis of variance. The relationship between miR-425-5p and RNF11 expressions was evaluated using Spearman's correlation analysis. The Kaplan–Meier and log-rank tests were used to assess RFS and DSS. The clinical prognostic significance of miR-425-5p was calculated by univariate and multivariate Cox regression analysis.

## 3. Results

### 3.1. miR-425-5p Was Upregulated in HCC Tissues

**Figure 1:**
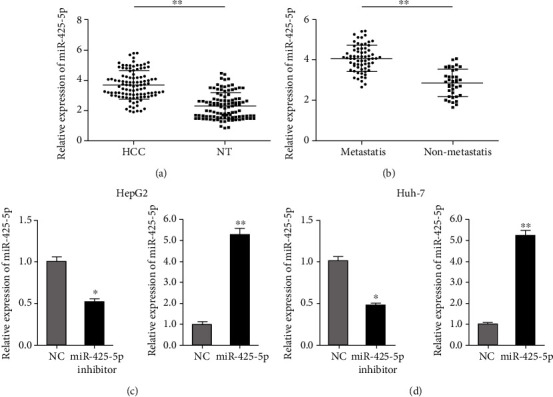
miR-425-5p was increased in the HCC tissues. (a) The expression of miR-425-5p in HCC tissue and paired adjacent tissue. (b) The expression of miR-425-5p in metastatic HCC and nonmetastatic HCC. (c) The expression of miR-425-5p in HepG2 cells with transfected miR-425-5p inhibitor and pre-miR-425-5p. (d) The expression of miR-425-5p in Huh-7 cells with transfected miR-425-5p inhibitor and pre-miR-425-5p. ^∗^*P* < 0.05, ^∗∗^*P* < 0.01.

To explore the role of miR-425-5p, RT-qPCR was performed to evaluate the expression of miR-425-5p in HCC. The results showed that miR-425-5p was significantly upregulated in HCC tissues compared with paired adjacent tissue (Figure 1(a)). Moreover, the expression of miR-425-5p in metastatic HCC is higher than that in nonmetastatic HCC (Figure 1(b)). To further explore the role of miR-425-5p in HCC, HepG2 and Huh-7 cells were transfected with pre-miR-425-5p to increase the expression of miR-425-5p, and miR-425-5p inhibitor to knockdown the expression of miR-425-5p (Figures 1(c) and 1(d)).

### 3.2. Overexpression of miR-425-5p Correlates with Aggressive Clinicopathological Features and Poor Prognosis of HCC

**Figure 2:**
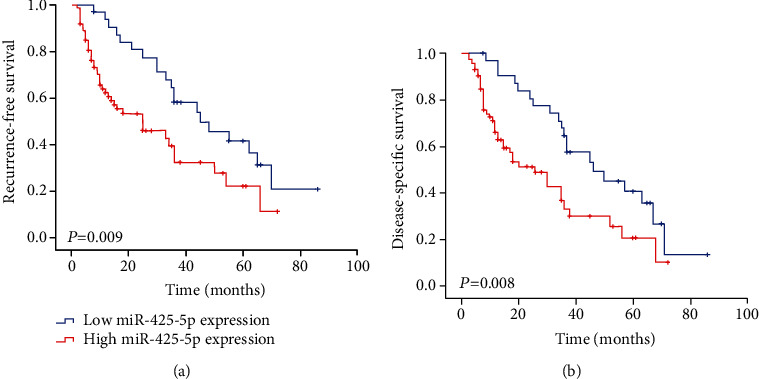
Kaplan–Meier survival curves of patients with miR-425-5p expression. (a) Kaplan–Meier analysis of RFS between HCC with high and low miR-425-5p expression. (b) Kaplan–Meier analysis of DSS between HCC with high and low miR-425-5p expression.

By analyzing the relationship between miR-425-5p and clinical prognosis of patients with HCC, we found that the overexpression of miR-425-5p expression was significantly correlated with TNM stage, vascular invasion, multiplicity, and intrahepatic metastasis (Table 1). Moreover, the Kaplan–Meier analysis showed that the patients with high miR-425-5p expression had a significantly shorter recurrence-free survival (RFS) than those with low miR-425-5p expression (Figure 2(a)). The TNM stage (*P* = 0.017), multiplicity (*P* = 0.024), vascular invasion (*P* = 0.010), intrahepatic metastasis (*P* = 0.028), and miR-425-5p (*P* = 0.011) were independent prognostic factors for RFS in HCC patients (Table 2).

We also analyzed the effect of miR-425-5p on disease-specific survival (DSS) in HCC. The Kaplan–Meier analysis showed that the patients with high miR-425-5p expression had a significantly shorter DSS than those with low miR-425-5p expression (Figure 2(b)). The TNM stage (*P* = 0.011), multiplicity (*P* = 0.015), vascular invasion (*P* = 0.025), intrahepatic metastasis (*P* = 0.038), and miR-425-5p (*P* = 0.010) were independent prognostic factors for DSS in HCC patients (Table 3).

### 3.3. Reducing the Expression of miR-425-5p Inhibits Cell Proliferation and Migration in HCC

**Figure 3:**
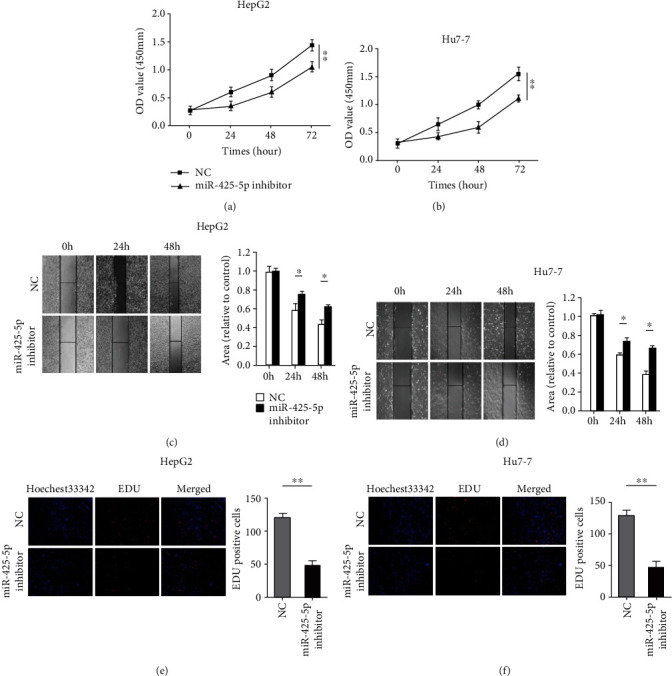
Decreased miR-425-5p inhibits the proliferation and migration of HCC. (a, b) The effect of miR-425-5p silent on the proliferation of HCC cells was analyzed using CCK-8 assays. (c) The wound healing assay was used to examine the effects of miR-425-5p silent on HepG2 cell migration. (d) The wound healing assay was used to examine the effects of miR-425-5p silent on Huh-7 cell migration. (e) Cell proliferation was detected with EDU in HepG2 cells after transfected with miR-425-5p inhibitor. (f) Cell proliferation was detected with EDU in Huh-7 cells after transfected with miR-425-5p inhibitor. ^∗^*P* < 0.05, ^∗∗^*P* < 0.011.

We used cell biology experiments to examine the role of miR-425-5p in HCC. CCK-8 assays showed that the decreased expression of miR-425-5p significantly inhibits the proliferation in HepG2 and Huh-7 cells (Figures 3(a) and 3(b)). Wound healing assays revealed that the decreased expression of miR-425-5p suppressed the migration of HepG2 and Huh-7 cells (Figures 3(c) and 3(d)). Moreover, HepG2 and Huh-7 cells incorporating EdU in the miR-425-5p downexpression group was less than the control group (Figures 3(e) and 3(f)).

### 3.4. miR-425-5p Directly Interacts with RNF11

**Figure 4:**
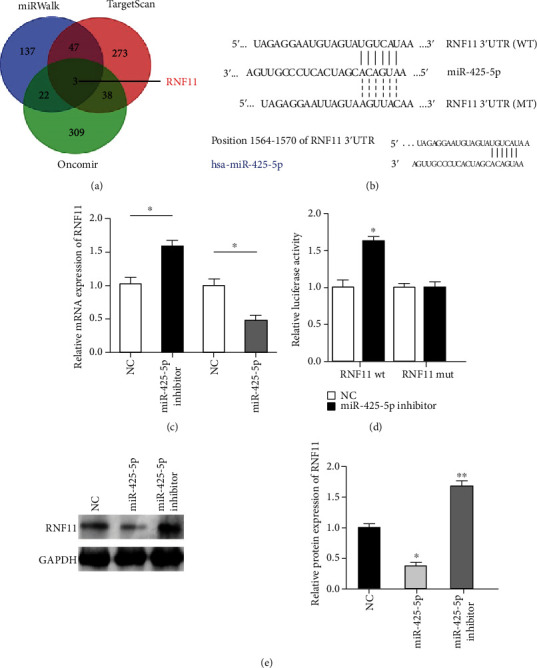
miR-425-5p directly targeted and regulated RNF11. (a) Venn diagram displaying miR-425-5p computationally predicted to target RNF11 by TargetScan, Oncomir, and miRWalk. (b) A schematic diagram of miR-425-5p putative binding sites and the corresponding mutant sites of RNF11. (c) RT-qPCR was used to examine the mRNA levels of RNF11 in HCC cells that were cotransfected with pre-miR-425-5p and miR-425-5p inhibitor. (d) The luciferase activity was used to detect the direct effect of miR-425-5p and RNF11. (e) Western blotting analysis was used to examine the protein expression of RNF11 in HCC cells that were cotransfected with pre- miR-425-5p and miR-425-5p inhibitor. ^∗^*P* < 0.05, ^∗∗^*P* < 0.01.

We examined miRNA databases (TargetScan, Oncomir, and miRWalk) and found that RNF11 was listed as a potential downstream target gene of miR-425-5p (Figures 4(a) and 4(b)). The decreasing of miR-425-5p significantly promoted both the mRNA and protein expressions of RNF11 (Figures 4(c) and 4(e)). Moreover, cotransfection of miR-425-5p inhibitor significantly increased luciferase activity in cells transfected with Wt RNF11 3′-UTR. In contrast, the growth phenomenon was not observed in cells cotransfected with Mt RNF11 3′-UTR (Figure 4(d)).

### 3.5. RNF11 Was Downregulated in HCC Tissue

**Figure 5:**
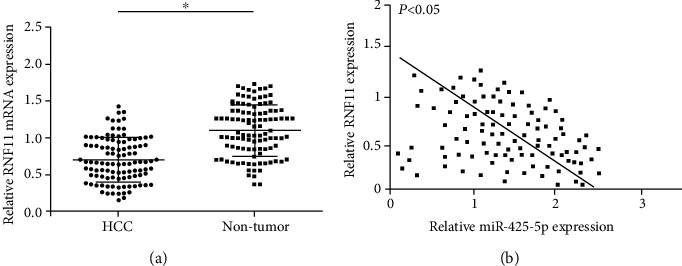
RNF11 decreased in HCC tissues. (a) The expression of RNF11 in HCC tissue and paired adjacent tissue. (b) miR-425-5p expression was negatively correlated with RNF11 expression in HCC. ^∗^*P* < 0.05.

Based on the above results, we further studied the expression of RNF11 in HCC. RT-qPCR results showed that RNF11 was downregulated in HCC tissues than that in paired adjacent tissue (Figure 5(a)). Importantly, miR-425-5p expression levels were inversely correlated with RNF11 (Figure 5(b)).

### 3.6. Restoration of RNF11 Rescued the miR-425-5p-Mediated Effect on Proliferation and Migration of HCC

**Figure 6:**
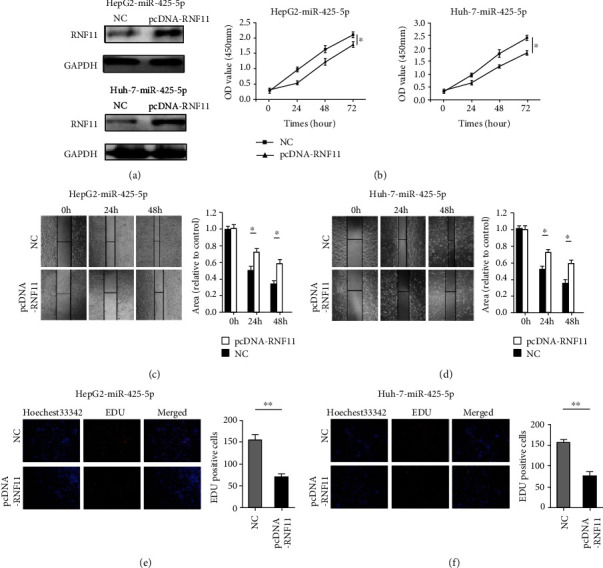
Restoring the expression of RNF11 rescued the miR-425-5p-mediated effect on HCC cells. (a) Western blot showed the expression of RNF11 in HepG2-miR-425-5p and Huh-7-miR-425-5p after overexpression of RNF11. (b) Effect of RNF11 on HepG2-miR-425-5p and Huh-7-miR-425-5p proliferation was analyzed using CCK-8 assays. (c) The wound healing assay was used to examine the effects of overexpression of RNF11 in HepG2- miR-425-5p. (d) The wound healing assay was used to examine the effects of overexpression of RNF11 in Huh-7-miR-425-5p. (e) Cell proliferation was detected with EDU in HepG2- miR-425-5p after overexpression of RNF11. (f) Cell proliferation was detected with EDU in Huh-7-miR-425-5p after overexpression of RNF11. ^∗^*P* < 0.05, ^∗∗^*P* < 0.01.

To explore whether miR-425-5p plays a biological role via RNF11 in HCC, we restored the expression of RNF11 by using RNF11 plasmids in HepG2 and Huh-7 cells, which overexpress miR-425-5p (Figure 6(a)). CCK-8 assays showed that the increased expression of RNF11 alleviates the promotion of miR-425-5p on the proliferation in HepG2 and Huh-7 cells (Figure 6(b)). Moreover, wound healing assays revealed that the increased expression of RNF11 alleviates the promotion of miR-425-5p on the migration in HepG2 and Huh-7 cells (Figures 6(c) and 6(d)). After increasing the expression of RNF11, the ability of HepG2 and Huh-7 cells to absorb EDU was limited (Figures 6(e) and 6(f)).

## 4. Discussion

HCC is one of the most common malignant tumors in China [24]. The mortality rate ranks third among malignant tumors of the digestive system. About 110,000 people die of HCC every year in China, accounting for 45% of the world's HCC deaths [25]. At present, many oncogenes are known to induce or promote the metastatic potential of HCC cells, such as Ras, Myc, Raf, Fos, EGFR, and c-Met, while KAI1 and PTEN can inhibit the metastasis and recurrence of cancer [26, 27]. Recent research found that microRNA (miRNA) is a kind of endogenous noncoding RNA with regulatory function found in eukaryotes. The main biological function of mature miRNA is to downregulate gene expression by inhibiting mRNA translation and promoting mRNA degradation [28]. However, the current research on microRNAs in HCC mainly focuses on the microRNAs with high expression in cancer tissues, such as miR-21-5p, miR-107, and miR-92a, or the microRNAs with low expression in advanced liver cancer, such as miR-122, miR-7, and miR-29a [29, 30]. Whether the change of miR-425-5p expression is related to the malignant behavior of HCC cells and whether the downstream molecules play an important role in the occurrence and development of HCC need further study. The purpose of this study is to explore the molecular mechanism of malignant development of HCC from the perspective of miR-425-5p and to provide a scientific basis for the treatment of patients with HCC.

The role of miRNA tumors has been widely recognized. As a functional microRNA, miR-425-5p plays different roles in tumors [31–33]. Recently, a study reported that miR-425-5p targeted genes are involved in the EGFR tyrosine kinase inhibitor resistance pathway, and miR-425-5p might act as an oncogene to participate in the pathogenesis of KRAS-mutated CRC [34]. miR-425-5p as a tumor suppressor gene is low expressed in prostate cancer and can affect the sensitivity of prostate cancer to cisplatin through the action of GSK3*β* and Wnt/*β*-catenin signaling pathways [35]. It has been found that long noncoding RNA LINC-PINT regulates laryngeal carcinoma cell stemness and chemoresistance by targeting miR-425-5p in laryngeal tumors [36]. Our results are also consistent with previous researches; miR-425-5p is highly expressed in HCC and is closely related to the poor prognosis of HCC patients. The inhibition of miR-425-5p expression can significantly control the malignant development of HCC via RNF11. These findings, combined with our existing results, suggest that miR-425-5p might play different roles in different tumor microenvironments. This is a very interesting phenomenon; we will explore the mechanism that may cause the above arrears in the next study.

RNF11 is a member of the ubiquitin ligase family and capable of modulating protein function through ubiquitination and perhaps through sumoylation, which may play an important role in tumor formation [37, 38]. RNF11 is abnormally expressed in breast cancer and can promote the malignant development of breast cancer [39]. In addition, RNF11 is activated by ETS1 and plays an important role in the growth of bone cells [40]. Moreover, RNF11 can directly enhance TGFb signaling through a direct association with SMAD4 and promote the malignant development of tumor cells [18]. Ubiquitination is activated by ubiquitination activator E1 in the form of ATP, and the activated ubiquitin protein is transported to ubiquitination polymerase E2, and then interacts with special E3 ubiquitin linker, resulting in protein ubiquitination. Importantly, RNF11 can interact with E2 binding enzyme and E3 ubiquitin ligase to promote protein ubiquitination [41]. In addition, RNF11 enhances the signal transduction of TGF-*β* at the receptor level. RNF11 directly connects with SMAD4. SMAD4 is a common SMAD that interacts with TGF-*β*. RNF11 can enhance the transcriptional activity of SMAD4 and SMAD2-SMAD4 transcriptional complexes, thus playing an important role in regulating the pathway [42]. The research results also showed that RNF11 could regulate the single phosphorylation of ESP-15 to degrade AMSH and regulate the ubiquitination of ESP-15, thus promoting the degradation of EGFR [20]. According to our research results and previous literature reports, we hypothesized that miR-425-5p affects the ubiquitination modification by regulating the expression of RNF11, which weakens the binding of RNF11 to E2 binding enzyme and E3 ubiquitination ligase, and then affects the malignant biological behavior of HCC by regulating TGF-*β* and EGFR signaling pathway. In the present work, we demonstrated that the high expression of miR-425-5p leads to the dysfunction of RNF11, and promoting the development of HCC. Therefore, through clinical samples and biological function experiments, we have determined that RNF11 as a new target for miR-425-5p to regulate tumorigenesis and development in HCC.

## 5. Conclusion

In general, miR-425-5p is highly expressed in HCC and is associated with poor prognosis. Reducing the expression of miR-425-5p can inhibit the proliferation and migration of HCC. Moreover, it is verified that RNF11 is a new downstream target of miR-425-5p. These findings will provide a new target for the diagnosis and treatment of HCC and provide a scientific basis for the further study of miR-425-5p in HCC.

## Figures and Tables

**Table 1:** Correlations between miR-425-5p and clinicopathological features of HCC patients.

Variables	Cases	miR-425-5p expression		*P*
		Low (*n* = 32)	High (*n* = 73)	
Age (yr)				
<50	48	15	33	0.874
≥50	57	17	40	
Gender				
Male	60	19	41	0.760
Female	45	13	32	
Tumor size (cm)				
≤5	50	14	36	0.599
>5	55	18	37	
AFP (ng/ml)				
≤20	38	12	26	0.853
>20	67	20	47	
TNM stage				
I/II	47	20	27	0.016
III/IV	58	12	46	
Liver cirrhosis				
Presence	57	19	38	0.488
Absence	48	13	35	
HBsAg				
Positive	65	21	44	0.603
Negative	40	11	29	
Vascular invasion				
Presence	55	10	45	0.004
Absence	50	22	28	
Multiplicity				
Single	42	18	24	0.024
Multiple (≥2)	63	14	49	
Intrahepatic metastasis				
Presence	52	11	41	0.040
Absence	53	21	32	

**Table 2:** Univariate and multivariate analysis of different prognostic variables influencing RFS in HCC patients.

Variables	*n*	Univariate analysis		Multivariate analysis model	
		HR (95% CI)	*P*	HR (95% CI)	*P*
Gender		0.348 (0.475-2.047)	0.640		
Male	60				
Female	45				
Age (year)		0.547 (0.308-1.571)	0.834		
<50	48				
≥50	57				
Tumor size (cm)		1.184 (0.674-1.876)	0.374		
≤5	50				
>5	55				
AFP(ng/ml)		1.037 (0.408-1.573)	0.941		
≤20	38				
>20	67				
HBsAg		0.768 (1.270-3.317)	0.329		
Positive	65				
Negative	40				
TNM stage		1.204 (1.743-3.269)	0.009	1.334 (1.278-3.004)	0.017
I/II	47				
III/IV	58				
Multiplicity		1.427 (1.127-3.690)	0.022	1.409 (1.045-3.347)	0.024
Single	42				
Multiple (≥2)	63				
Vascular invasion		1.894 (1.806-5.974)	0.014	1.743 (1.874-4.339)	0.010
Presence	55				
Absence	50				
Liver cirrhosis		0.884 (0.474-1.634)	0.735		
Presence	57				
Absence	48				
Intrahepatic metastasis		0.986 (0.640-2.047)	0.033	1.228 (1.475-3.993)	0.028
Presence	52				
Absence	53				
miR-425-5p expression		1.220 (0.471-1.649)	0.017	1.171 (0.503-1.843)	0.011
High	73				
Low	32				

HR: hazard rate; CI: confidence interval.

**Table 3:** Univariate and multivariate analysis of different prognostic variables influencing DSS in HCC patients.

Variables	*n*	Univariate analysis		Multivariate analysis model	
		HR (95% CI)	*P*	HR (95% CI)	*P*
Gender		0.535 (0.137-1.087)	0.370		
Male	60				
Female	45				
Age (year)		0.472 (0.634-1.871)	0.476		
<50	48				
≥50	57				
Tumor size (cm)		0.807 (0.934-1.997)	0.710		
≤5	50				
>5	55				
AFP (ng/ml)		1.343 (0.841-1.947)	0.841		
≤20	38				
>20	67				
HBsAg		1.005 (0.475-2.614)	0.934		
Positive	65				
Negative	40				
TNM stage		1.374 (1.057-3.004)	0.019	1.201 (1.847-4.647)	0.011
I/II	47				
III/IV	58				
Multiplicity		1.340 (0.543-1.670)	0.012	1.284 (0.843-1.842)	0.015
Single	42				
Multiple (≥2)	63				
Vascular invasion		1.556 (1.706-2.648)	0.030	1.408 (1.641-2.330)	0.025
Presence	55				
Absence	50				
Liver cirrhosis		1.109 (0.847-1.739)	0.535		
Presence	57				
Absence	48				
Intrahepatic metastasis		0.884 (1.045-2.984)	0.043	1.047 (2.034-5.376)	0.038
Presence	52				
Absence	53				
miR-425-5p expression		1.469 (0.567-1.886)	0.007	1.338 (0.807-2.364)	0.010
High	73				
Low	32				

HR: hazard rate; CI: confidence interval.

## Data Availability

The data used to support the findings of this study are available from the corresponding author upon request.
